# Endothelial DR6 in blood-brain barrier malfunction in Alzheimer’s disease

**DOI:** 10.1038/s41419-024-06639-0

**Published:** 2024-04-12

**Authors:** Xiaomin Huang, Junhua Qi, Yixun Su, Ying Zhou, Qi Wang, Taida Huang, Dongdong Xue, Yunxin Zeng, Alexei Verkhratsky, Benjie Zhou, Hui Chen, Chenju Yi

**Affiliations:** 1 Research Centre, Seventh Affiliated Hospital of Sun Yat-sen University, Shenzhen, China; 2 The Brain Cognition and Brain Disease Institute, Shenzhen Institutes of Advanced Technology, Chinese Academy of Sciences, Shenzhen, 518055 Guangdong China; 3 Faculty of Biology, Medicine and Health, The University of Manchester, Manchester, UK; 4 Achucarro Center for Neuroscience, IKERBASQUE, Bilbao, Spain; 5 Department of Stem Cell Biology, State Research Institute Centre for Innovative Medicine, Vilnius, Lithuania; 6 Department of Forensic Analytical Toxicology, School of Forensic Medicine, China Medical University, Shenyang, China; 7 Department of Pharmacy, The Seventh Affiliated Hospital of Sun Yat-sen University, Shenzhen, China; 8 Shenzhen Key Laboratory of Chinese Medicine Active Substance Screening and Translational Research, Shenzhen, 518107 China; 9 School of Life Sciences, Faculty of Science, University of Technology Sydney, Ultimo, NSW 2007 Australia; 10 Guangdong Provincial Key Laboratory of Brain Function and Disease, Guangzhou, China

**Keywords:** Blood-brain barrier, Alzheimer's disease

## Abstract

The impairment of the blood-brain barrier (BBB) has been increasingly recognised as a critical element in the early pathogenesis of Alzheimer’s disease (AD), prompting a focus on brain endothelial cells (BECs), which serve as the primary constituents of the BBB. Death receptor 6 (DR6) is highly expressed in brain vasculature and acts downstream of the Wnt/β-catenin pathway to promote BBB formation during development. Here, we found that brain endothelial DR6 levels were significantly reduced in a murine model of AD (APP_swe_/PS1_dE9_ mice) at the onset of amyloid-β (Aβ) accumulation. Toxic Aβ_25-35_ oligomer treatment recapitulated the reduced DR6 in cultured BECs. We further showed that suppressing DR6 resulted in BBB malfunction in the presence of Aβ_25-35_ oligomers. In contrast, overexpressing DR6 increased the level of BBB functional proteins through the activation of the Wnt/β-catenin and JNK pathways. More importantly, DR6 overexpression in BECs was sufficient to rescue BBB dysfunction in vitro. In conclusion, our findings provide new insight into the role of endothelial DR6 in AD pathogenesis, highlighting its potential as a therapeutic target to tackle BBB dysfunction in early-stage AD progression.

## Introduction

Alzheimer’s disease (AD) poses significant challenges to neuroscience due to the complexity of its pathophysiology [1]. Because amyloid-β (Aβ) plaques are commonly recognised as the primary indicator and fundamental mechanism of neurodegeneration associated with AD, existing treatments primarily target the removal of Aβ aggregates. However, these treatments are typically prescribed to individuals in the early stages of AD and do not yield substantial enhancements in cognitive functions [2]. Therefore, the development of new therapeutics is in demand. These new strategies are most likely connected with mechanisms distinct from Aβ deposition. In particular, the impairment of the blood-brain barrier (BBB) is increasingly recognised as a critical factor in the early stages of AD pathogenesis [3, 4]. The BBB primarily consists of brain endothelial cells (BECs) associated with a layer of vascular and parenchymal basement membranes, along with pericytes and astrocyte endfeet. Together, these cells create a selective barrier that separates the bloodstream from the brain parenchyma, facilitating controlled transport of substances into and out of the brain [5]. The findings on BBB malfunction from AD patients and studies using animal models are consistent, which all showed impaired ability to selectively transport molecules across BBB, as well as compromised barrier function of BBB [3]. These disruptions have the potential to disturb brain homeostasis and exacerbate the pathology associated with AD.

As the core components of the BBB, BECs receive increasing attention in the context of AD pathogenesis. Significant alterations in the endothelial transcriptome have been found in AD patients, which demonstrated the prevalent expression of most AD-related risk genes within the brain vasculature [6]. Targeting BECs presents a promising therapeutic avenue for AD management. However, such an approach requires a better understanding of pathophysiological changes in the vascular system in AD. Previously, we showed that toxic Aβ oligomers inhibit the endothelial Wnt/β-catenin pathway in brain microvessels with subsequent suppression of tight junction proteins, leading to impaired BBB functions [4]. However, a comprehensive understanding of the downstream regulatory network governing endothelial Wnt/β-catenin signalling warrants further investigation.

Death receptor 6 (DR6, encoded by *Tnfrsf21*), a member of the tumour necrosis factor receptor family, acts downstream of the Wnt/β-catenin pathway during the development of the central nervous system (CNS) [7]. This is because DR6 is enriched in the vasculature and is essential for angiogenesis, proper vascular structure in the CNS, and BBB functions [7]. Total DR6 levels are increased in the cortex and hippocampus of APP/PS1 mice and in the cortex of AD patients. Previous studies mainly focused on the role of DR6 on neuronal health, reflecting the interaction between Aβ precursor protein (APP) and DR6. Specifically, DR6 is the receptor of the amino-terminus of APP (N-APP), binding of which results in axon pruning and neuronal death [8–11]. In vitro studies showed that Aβ can induce N-APP release and increase DR6 levels, which exacerbate neuronal death [10, 11]. Therefore, neuronal DR6 has been suggested to contribute to neurodegeneration in AD pathology [7, 12, 13]. However, global DR6 knockout did not reverse the pathological outcome in AD mice [10]. On the contrary, global DR6 knockout in wild-type mice induced a similar phenotype to that in AD mice [10]. The latter observations suggest that DR6 in non-neuronal cells may have an opposite role in the pathogenesis of AD to that in neurons, and the effect is perhaps more prominent than its neuronal form.

Based on the observations that DR6 knockout reduced BBB functional protein glucose transporter 1 (Glut-1) and caused BBB leakage in mice and zebrafish [7], we hypothesised that BEC DR6 may play a protective role in maintaining BBB integrity and function in the presence of Aβ; while impairments of BEC DR6 can occur during AD pathogenesis. In this study, we isolated BECs for in vitro experiments. In response to Aβ, the DR6 level was reduced in BECs, along with reduced Wnt and JNK pathway signalling elements. Subsequently, we employed lentivirus techniques to knockdown and overexpress DR6 in BECs to determine whether these approaches can change the responses of Wnt and JNK signalling to Aβ-induced toxicity in BECs.

## Materials and methods

### Animal model of AD

All animal experiments were approved by the Institutional Animal Care and Use Committee, Shenzhen Bay Laboratory (Approval# IACUC- AEYCJ202202) and performed in compliance with the Guide for the Care and Use of Laboratory Animals. Swedish mutant APP (APP695_swe_)/PS1 (PSEN1dE9) transgenic mice (2, 4, 9, and 12 months old) and age-matched C57BL/6J wild-type (WT) mice (Junke Co., Ltd., Nanjing, China) were housed in a pathogen-free facility under 22 ± 1 °C, 50 ± 10% humidity, and 12 h light/12 h dark cycles, with *ad libitum* access to standard rodent chow and water. Both males and females were used.

### Primary BECs culture and lentivirus transfection

BECs were isolated as previously described [14]. Briefly, the cortices of 3-month-old WT mice were collected and incubated in 10 mL of DMEM containing 1 mg/mL collagenase (Sigma-Aldrich, C6885) and 0.1 mg/mL DNase I (Sigma-Aldrich, DN25) at 37 °C for 1 h. Then, the homogenates were centrifuged at 1000 g for 8 min at room temperature. The pellet was suspended with 25 ml 20% BSA-DMEM and centrifugated at 1000 g for 20 min. The resulting microvessel pellet was then homogenised in DMEM containing 1 mg/mL collagenase-dispase (Sigma-Aldrich, 10269638001) and 0.1 mg/mL DNAse I and incubated at 37 °C for 45 min. After centrifugation (1000 rpm for 6 min), the cell pellet was resuspended in the BECs culture medium, followed by seeding on collagen-coated plates. Puromycin was supplied in the medium for the first day to achieve a high BECs purity. When 40–50% confluence was reached, cells were transfected with empty carrier Lentivirus (Lenti-Ctrl, OBiO Technology (Shanghai) Corp., Ltd) or pSLenti-EF1-EGFP-P2A-Puro-CMV-Tnfrsf21-3xFLAG-WPRE (Lenti-DR6) and pSLenti-U6-shRNA (Tnfrsf21)-CMV-EGFP-F2A-Puro-WPRE (Lenti-shDR6) for 72 h and ready for subsequent experiments.

### Aβ oligomers treatment

Aβ oligomers were prepared as previously described [4]. Briefly, synthetic Aβ_25−35_ peptide (Sigma-Aldrich, A4559) was diluted in dimethyl sulfoxide (Sigma-Aldrich, D2650) and lyophilised overnight. To form Aβ_25__–__3__5_ oligomers, the lyophilised stock was resuspended in culture medium to the desired concentration and incubated at 37 °C for 24 h before the experiments. BECs were treated with Aβ_25–35_ at 10 μM for 24 h after transduced by lentivirus for 48 h. At least three independent experiments were performed.

### Immunofluorescence staining

After deep anaesthesia (1% tribromoethanol), mice were perfused with cold PBS and 4% paraformaldehyde. Primary BECs cultured on coverslips were fixed with 4% paraformaldehyde for 15 min. The brains were dehydrated, embedded in OCT media, and snap frozen. Frozen brain sections (20 μm) and BECs were incubated with blocking buffer (PBS containing 0.2% gelatin and 0.5% Triton-X-100) for 30 min at room temperature. After blocking, sections or coverslips were incubated at 4 °C overnight with primary antibodies (CD31 (1:200, rat, 553370, BD), fibrinogen (1:500, mouse, ab58207, Abcam), DR6 (1:100, rabbit, bs-7678R, Bioss), claudin-5 (Cldn-5) (1:100, rabbit, 35-2500, Invitrogen), Glut-1 (1:200, rabbit, HPA031345, Sigma), zonula occludens (Zo)-1 (1:200, mouse, 339100, Invitrogen)), followed by secondary antibodies (Alexa Fluor 488 (1:1000), Alexa Fluor 555 (1:1000) or/and Alexa Fluor 647 (1:1000), Thermo Fisher Scientific) for 2 h at room temperature. Then, the sections or coverslips were incubated with DAPI for 10 min, mounted and assessed with a confocal microscope (Plan-Apochromat 63 Oil DIC M27 objective (Zeiss) for CD31 co-staining with DR6, UPLXAPO 40x magnification (Olympus) for CD31 co-staining with fibrinogen/Cldn-5/Glut-1 sections, or slice scanner VS200 (Olympus) for coverslips). Six non-overlapping slices from each brain were analysed using Fiji software (NIH, USA). CD31 co-staining was used to show target markers specifically in blood vessels. All results were normalised to the average value of the WT group. For the experiments on BECs, three independent experiments were performed in triplicates. Five non-overlapping areas from each image were analysed. The fluorescence density was calculated by Fiji software, normalised to the control group, and presented as a ratio.

### Western blotting

Cells were lysed in RIPA lysis buffer (Epizyme, PC101) supplemented with a protease/phosphatase inhibitor cocktail. Proteins were quantified by BCA Protein Assay Kit (Epizyme, ZJ101), supplemented with the loading buffer, and placed in a metal bath at 95 °C for 10 min. After being separated by SDS–polyacrylamide gel electrophoresis, proteins were transferred to PVDF membranes and blocked in PBS containing 5% BSA and 0.1% Tween20. Then, membranes were incubated with primary antibodies (DR6 (1:100, rabbit, bs-7678R, Bioss), Cldn-5 (1:1000, rabbit, AF5216, Affinity), Glut-1 (1:1000, rabbit, ab115730, Abcam), active β-catenin (1:1000, rabbit, 8814, CST), total β-catenin (1:1000, rabbit, 8480, CST), Phospho-JNK (1:1000, rabbit, AP1337, Abclonal), JNK (1:1000, rabbit, A4867, Abclonal) and HRP-Conjugated GAPDH (1:10,000, HRP-60004, Proteintech)) overnight at 4 °C, followed by corresponding secondary antibodies, and detected with SuperSignal West Atto Ultimate Sensitivity Substrate (Thermo Fisher Scientific, A38555) by ChemiDoc MP Imaging System (Bio-Rad, USA). Fiji software was used to quantify band densities. The quantification of phosphorylated proteins was normalised to their total proteins. GAPDH was used as the housekeeping protein, and the data were presented as fold change to the control group.

### Real-time PCR

Total RNA was extracted by RNAzol reagent (Sigma-Aldrich, R4533) and cDNA was synthesised with Evo M-MLV reverse transcriptase kit (Accurate Biology, AG11706) according to the manufacturer’s instructions. Real-time PCR was carried out using SYBR Green Premix Pro Taq HS qPCR Kit (Accurate Biology, AG11701). The primers are listed in Supplementary Table 1. The relative expression of target genes was calculated using the 2^−ΔΔCt^ method normalised to the housekeeping gene *Gapdh*. The results are presented as a fold change compared to the controls.

### Trans-well permeability assay

Primary BECs were seeded on a 24-well plate with a 0.4 μm pore polycarbonate membrane inserted into a trans-well plate. After the desired treatments, the cells were subjected to a permeability assay. The upper chamber was filled with 150 μL of 1 mg/mL FITC-dextran (40 kDa), while the bottom well was replaced with 600 μL of fresh BEC culture media. Samples were collected from the bottom well at 30 min. Fluorescence intensities were measured at 485 nm excitation and 535 nm emission wavelength. The data were normalised to the control group.

### Statistical analysis

Data are presented as mean ± standard deviation (SD). For two groups, data were analysed by a two-tailed Student’s *t* test; for >2 groups, data were analysed by one-way ANOVA followed by Tukey post hoc tests (GraphPad Prism 9). *p* < 0.05 was considered statistically significant.

## Results

### BBB malfunction and reduced vascular DR6 levels in the hippocampus of APP/PS1 mice

**Fig. 1:**
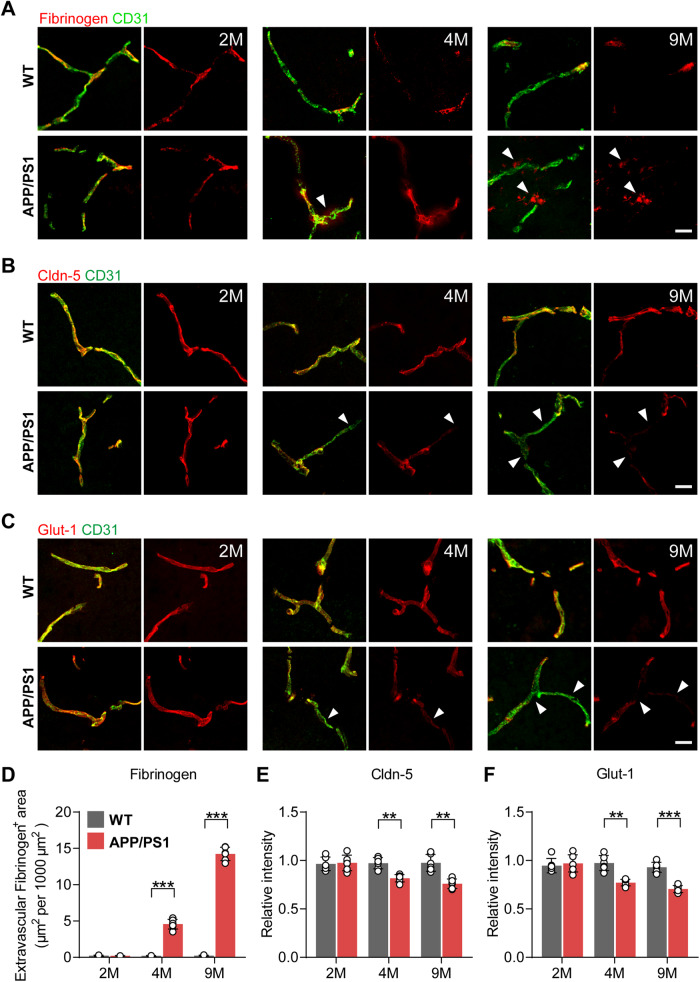
BBB malfunction and endothelial cell disruption in the hippocampus of APP/PS1 mice. Fibrinogen (**A**, **D**), Cldn-5 (**B**, **E**) and glucose transporter 1 (Glut-1) (**C**, **F**) co-staining with CD31 in the hippocampus of APP/PS1 mice and age-matched WT mice at 2, 4 and 9 months of age (scale bar = 20 µm). Data are presented as mean ± SD, *n* = 6 mice/group, *p < 0.05, **p < 0.01, ***p < 0.001 compared with age-matched WT mice.

We first confirmed the BBB leakage in AD mice using fibrinogen as a surrogate marker. In control WT mice, fibrinogen staining was restricted to the vascular lumen, suggesting intact BBB functions (Fig. 1A, D). In 2-month-old APP/PS1 mice, no extravascular accumulation of fibrinogen was observed in the brain parenchyma (*p* = 0.7361, Fig. 1A, D). In 4-month-old APP/PS1 mice, a significant extravascular accumulation of fibrinogen in the brain parenchyma was observed (*p* = 0.0004, Fig. 1A, D), consistent with BBB leakage at the early stages of AD in humans. This BBB leakage was further increased in 9-month-old APP/PS1 mice (*p* = 0.00001, Fig. 1A, D). Tight junction protein Cldn-5 and glucose transporter Glut-1 are essential for maintaining BECs’ integrity and BBB functions, which were significantly and steadily decreased in APP/PS1 mice since 4 months of age compared to age-matched WT mice (Cldn-5: 2 months, *p* = 0.8227; 4 months, *p* = 0.0035; 9 months, *p* = 0.0027. Glut-1: 2 months, *p* = 0.6339; 4 months, *p* = 0.0017; 9 months, *p* = 0.0005) (Fig. 1B, C, E, F).

**Fig. 2:**
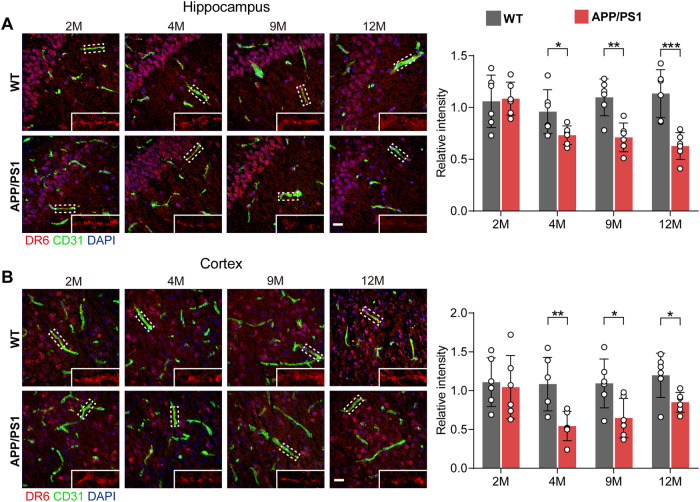
Reduced vascular DR6 levels in the APP/PS1 brains. Vascular DR6/CD31 co-staining in the hippocampus (**A**) and cortex (**B**) of APP/PS1 and age-matched WT mice at 2, 4, 9, and 12 months (scale bar = 20 µm). Data are presented as mean ± SD, *n* = 6 mice/group, *p < 0.05, **p < 0.01, ***p < 0.001 compared with age-matched WT mice.

As we previously showed that Wnt/β-catenin signalling is reduced in BECs in APP/PS1 mice [4], while DR6 lies downstream of Wnt/β-catenin signalling [7], we investigated whether DR6 is also reduced in the brain endothelium of APP/PS1 mice. Indeed, immunostaining showed that DR6 levels in the blood vessels were significantly and continuously decreased in the hippocampus (2 months, *p* = 0.8421; 4 months, *p* = 0.0359; 9 months, *p* = 0.0018; 12 months, *p* = 0.0008, Fig. 2A), and consistently decreased in the cortex (2 months, *p* = 0.7664; 4 months, *p* = 0.0071; 9 months, *p* = 0.0228; 12 months, *p* = 0.0215, Fig. 2B) of APP/PS1 mice from 4 months of age. These data suggest that the decreased vascular DR6 levels correlate with BECs impairment and BBB dysfunction in APP/PS1 mice.

### Aβ treatment impaired BECs and reduced DR6 levels in vitro

**Fig. 3:**
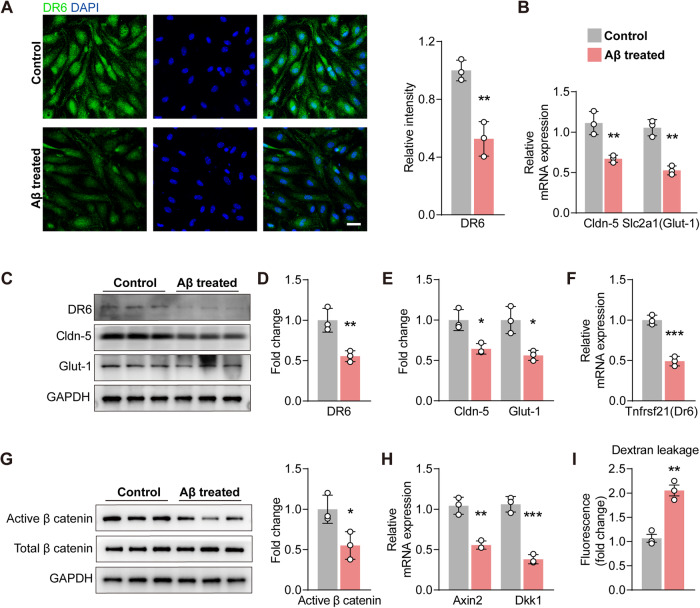
Aβ_25-35_ significantly reduced DR6 levels in BECs and suppressed BBB-related proteins in vitro. **A** Immunostaining and quantification of DR6 in BECs (scale bar = 25 µm); **B**, **F** mRNA expression of *Cldn-5*, *Slc2a1(Glut-1)*, and *Tnfrsf21(Dr6)*; **C**–**E** Protein levels of Cldn-5, Glut-1, and DR6; **G** Protein levels of active β-catenin; **H** mRNA expression of *Axin2* and *Dkk1*; **I** trans-well permeability assay. Data are presented as mean ± SD, *n* = 3 independent experiments/group, *p < 0.05, **p < 0.01.

To examine whether Aβ pathology is sufficient to induce endothelial DR6 reduction, we treated mouse primary cortical BECs with Aβ_25-35_ oligomers. The immunoreactivity of DR6 was significantly reduced in Aβ_25-35_ treated BECs (*p* = 0.0042, Fig. 3A). Changes in vascular DR6 level were further corroborated by measuring protein levels using western blotting (*p* = 0.0089) and mRNA expression using RT-PCR (*p* = 0.0005) (Fig. 3C, D, F). The alteration of DR6 expression in BECs in vitro is consistent with that in APP/PS1 mice in vivo, further suggesting that the Aβ pathology suppresses endothelial DR6 expression. Conspicuously in BECs, Aβ_25-35_ significantly decreased mRNA expression (*Cldn-5*, *p* = 0.0070; *Slc2a1(Glut-1)*, *p* = 0.0014) and protein levels of Cldn-5 (*p* = 0.0134) and Glut-1 (*p* = 0.0130) in BECs in vitro (Fig. 3B, C, E), consistent with our previous findings [4]. Functional disruption of the barrier by Aβ_25-35_ was demonstrated by analysing the permeability of BEC monolayers (*p* = 0.0021) (Fig. 3I). Furthermore, there was a marked reduction in active β-catenin protein levels (*p* = 0.0337, Fig. 3G) and mRNA expression of Wnt/ß-catenin signalling pathway scaffold protein axis inhibition protein 2 (*Axin2*, *p* = 0.0019) and ligand Dickkopf-1 (*Dkk1*, *p* = 0.0005) (Fig. 3H), also consistent with our previous findings [4]. These data suggest under the influence of Aβ, the suppression of DR6 correlates with BBB dysfunction.

### Downregulation of DR6 leads to BEC malfunction

**Fig. 4:**
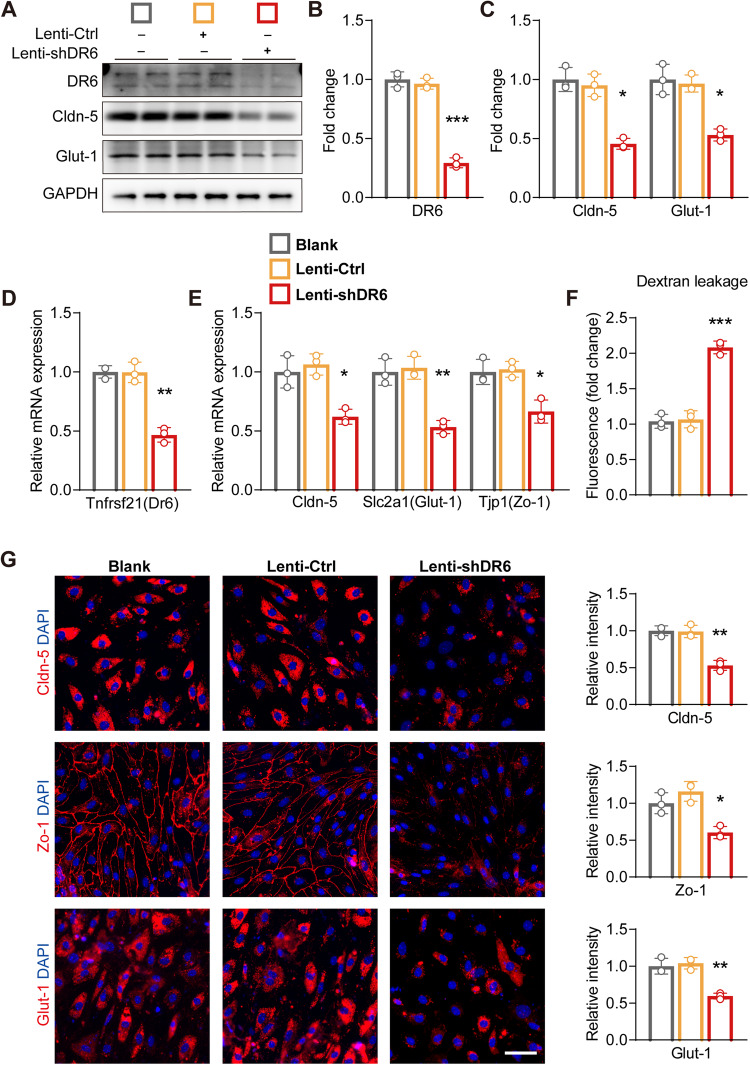
DR6 downregulation induced BEC disruption. **A**–**C** Protein levels of DR6, Cldn-5 and Glut-1; **D**, **E** mRNA expression of *Tnfrsf21(Dr6)*, *Cldn-5*, *Slc2a1(Glut-1)*, and *Tjp1(Zo-1)*; **F** In vitro trans-well permeability assay; **G** Immunostaining and quantification of Cldn-5, Zo-1 and Glut-1 in BECs (scale bar = 50 µm); Data are presented as mean ± SD, *n* = 3 independent experiments/group, *p < 0.05, **p < 0.01, ***p < 0.001.

To investigate whether the downregulation of DR6 instigates malfunction of the brain endothelium, we used lentivirus to knockdown DR6 in BECs. Compared to the non-infected control and the scramble shRNA control, DR6 knockdown resulted in a significant reduction in DR6 levels in BECs as shown by its protein level (*p* = 0.0001) and mRNA expression (*p* = 0.0031) (Fig. 4A, B, D). DR6 downregulation in BECs resulted in a marked decrease in immunoreactivity of Cldn-5 (*p* = 0.0010), Zo-1 (*p* = 0.0138), and Glut-1 (*p* = 0.0036, Fig. 4G). Changes in Cldn-5 and Glut-1 were further confirmed by protein levels using western blotting (Cldn-5, *p* = 0.0011; Glut-1, *p* = 0.0042; Fig. 4A, C) and mRNA expression (*Cldn-5*, *p* = 0.0122; *Glut-1*, *p* = 0.0030; *Tjp1(Zo-1)*, *p* = 0.0160, Fig. 4E). To examine whether the brain endothelial barrier function is affected by DR6 downregulation, we performed the trans-well permeability assay, showing that indeed DR6 knockdown increased FITC-dextran leakage to the lower chamber, reflecting impaired barrier function of BECs (*p* = 0.0002) (Fig. 4F).

Next, we overexpressed DR6 in BECs using lentivirus to examine if it can promote BBB-related protein. However, despite the high efficiency of overexpressing DR6, as confirmed by its protein (*p* = 0.0003) and mRNA (*p* = 0.0001) levels (Supplementary Fig. S1A, B, D), there were no significant changes in immunoreactivity of Cldn-5 (*p* = 0.6665), Zo-1 (*p* = 0.8968), and Glut-1 (*p* = 0.3205) (Supplementary Fig. S1G), confirmed by western blotting (Cldn-5, *p* = 0.7168; Glut-1, *p* = 0.9241) and real-time PCR (*Cldn-5*, *p* = 0.2422; *Glut-1*, *p* = 0.9761; *Zo-1*, *p* = 0.4601) (Supplementary Fig. S1A, C, E). Also, DR6 overexpression does not affect the barrier integrity of BECs as measured by the trans-well assay (*p* = 0.5517) (Supplementary Fig. S1F). Taken together, these results indicate that DR6 is necessary for maintaining BECs barrier proteins and function; however, an unknown mechanism may exist to gatekeep the maximum effect of DR6 in regulating BBB functional protein, i.e., plateauing the response regardless of the rising amount of DR6.

### DR6 overexpression ameliorates BECs dysfunction induced by Aβ_25-35_

**Fig. 5:**
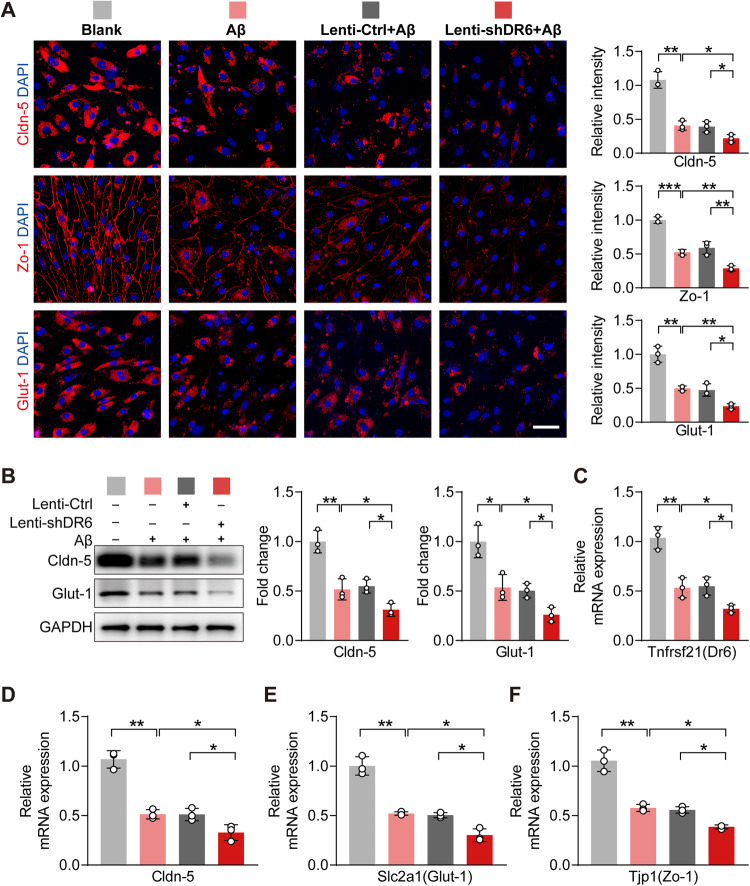
DR6 downregulation exacerbated Aβ_25–35_ induced BBB-related protein loss in BECs. **A** Immunostaining and quantification of Cldn-5, Zo-1, and Glut-1 in BECs (scale bar = 50 µm); **B** Protein levels of Cldn-5 and Glut-1; **C**–**F** mRNA expression of *Tnfrsf21(Dr6)*, *Cldn-5*, *Slc2a1(Glut-1)* and *Tjp1(Zo-1)*. Data are presented as mean ± SD, *n* = 3 independent experiments/group, *p < 0.05, **p < 0.01, ***p < 0.001.

To interrogate the role of BECs DR6 in AD pathology, we suppressed DR6 expression by lentivirus in BECs in vitro, followed by exposure to Aβ_25–35_ for 24 h. Real time-PCR confirmed the knockdown efficiency of *Tnfrsf21(Dr6)* shRNA in the presence of Aβ (*p* = 0.0145 versus Aβ_25–35_ condition and *p* = 0.0159 versus Lenti-Ctrl+Aβ) (Fig. 5C). Suppressing DR6 before Aβ_25–35_ treatment led to a further reduction in immunoreactivity of Cldn-5 (*p* = 0.0373 versus Lenti-Ctrl+Aβ), Zo-1 (*p* = 0.0066 versus Lenti-Ctrl+Aβ) and Glut-1 (*p* = 0.0137 versus Lenti-Ctrl+Aβ) (Fig. 5A). Western blot analysis confirmed that DR6 knockdown exacerbated the reduction in Cldn-5 (*p* = 0.0137) and Glut-1 (*p* = 0.0152) protein levels in the presence of Aβ_25–35_ (all versus Lenti-Ctrl+Aβ, Fig. 5B). There was also a reduction in mRNA expression of *Cldn-5* (*p* = 0.0351), *Glut-1* (*p* = 0.0174), and *Zo-1* (*p* = 0.0160) (all versus Lenti-Ctrl+Aβ, Fig. 5D–F). These results suggest suppressing DR6 can exacerbate Aβ_25–35_ induced BECs barrier protein impairment.

**Fig. 6:**
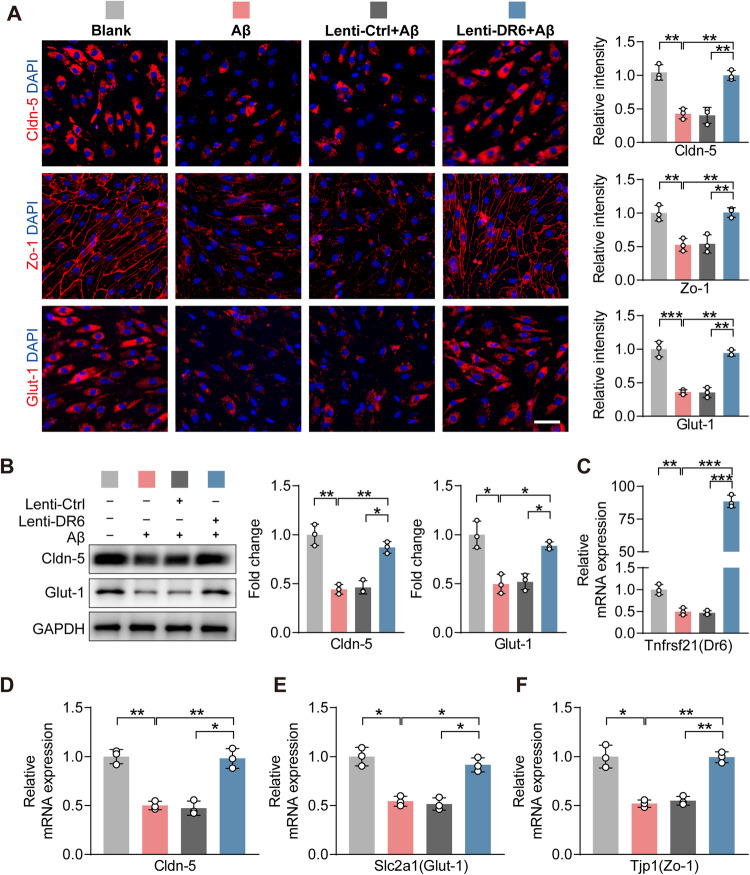
DR6 overexpression alleviated Aβ_25–35_-induced BEC malfunction. **A** Immunostaining and quantification of Cldn-5, Zo-1, and Glut-1 in BECs (scale bar = 50 µm); **B** Protein levels of Cldn-5 and Glut-1; **C**–**F** mRNA expression of *Tnfrsf21(Dr6)*, *Cldn-5*, *Slc2a1(Glut-1)*, and *Tjp1(Zo-1)*. Data are presented as mean ± SD, *n* = 3 independent experiments/group, *p < 0.05, **p < 0.01, ***p < 0.001.

To investigate whether increasing DR6 level can prevent Aβ-induced BECs functional impairment, we overexpressed DR6 in BECs in vitro, followed by Aβ_25–35_ treatment. The efficiency of overexpression was confirmed by *Dr6* mRNA levels (*p* = 5.59794 × 10^−6^, Lenti-DR6+Aβ versus Lenti-Ctrl+Aβ) (Fig. 6C). The immunoreactivities of Cldn-5, Zo-1, and Glut-1 were nearly normalised in DR6-overexpressed BECs when treated with Aβ_25–35_ (Cldn-5, *p* = 0.0019; Zo-1, *p* = 0.0022; Glut-1, *p* = 0.0012; all versus Lenti-Ctrl+Aβ) (Fig. 6A). Changes in Cldn-5 (*p* = 0.0154, versus Lenti-Ctrl+Aβ), and Glut-1 (*p* = 0.0210, versus Lenti-Ctrl+Aβ) were further confirmed by western blotting (Fig. 6B). mRNA levels of *Cldn-5* (*p* = 0.0215 versus Lenti-Ctrl+Aβ), *Glut-1* (*p* = 0.0195 versus Lenti-Ctrl+Aβ), and *Zo-1* (*p* = 0.0041 versus Lenti-Ctrl+Aβ) were also increased in DR6-overexpressed cells in response to Aβ_25-35_ treatment (Fig. 6D–F). These data suggest that overexpressing DR6 alleviates Aβ-induced impairment of BEC barrier-related proteins.

### DR6 alleviates Aβ_25–35_-induced BECs dysfunction through activation of Wnt and JNK signalling

**Fig. 7:**
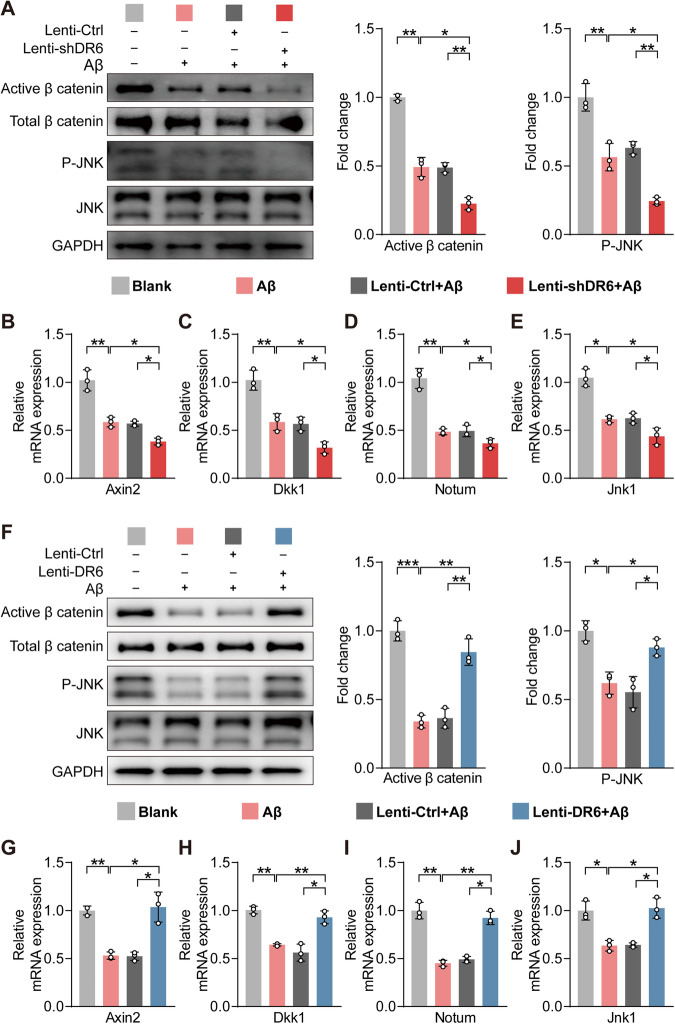
DR6 regulated BEC function through JNK signalling under Aβ_25–35_ treatment. **A**, **F** Protein levels of active β-catenin and phosphorylated JNK; **B**–**E**, **G**–**J** mRNA expression of *Axin2*, *Dkk1*, *Notum*, and *Jnk1*. Data are presented as mean ± SD, *n* = 3 independent experiments/group, *p < 0.05, **p < 0.01, ***p < 0.001.

Next, we investigated the mechanisms underlying the regulation of BECs barrier proteins by DR6. DR6 has been suggested to act through the JNK pathway to promote endothelial cell sprouting during brain angiogenesis; DR6 knockout reduced the expression of Wnt/β-catenin target genes [7]. Here, Aβ_25-35_ treatment suppressed Wnt/β-catenin and JNK signalling (Fig. 7A–F and Supplementary Fig. S2A) as indicated by reduced levels of active β-catenin (*p* = 0.0030 versus blank control) and phosphorylated JNK (*p* = 0.0061 versus blank control) (Fig. 7A and Supplementary Fig. S2A), as well as the mRNA expression of their target genes: *Axin2* (*p* = 0.0035), *Dkk1* (*p* = 0.0051), and the palmitoleoyl-protein carboxylesterase *Notum* (*p* = 0.0012) in the Wnt/β-catenin pathway and *Jnk1* (*p* = 0.0016) in the JNK pathway (all versus blank control, Fig. 7B–E).

As the activation of Wnt/JNK signalling correlates with the level of DR6, we investigated whether DR6 regulates the activities of this pathway. Knockdown of DR6 in the BECs in control conditions significantly reduced the activation of the Wnt/β-catenin pathway, as indicated by a lower level of active β-catenin (*p* = 0.0090) and a lower expression of Wnt/β-catenin pathway target genes, such as *Axin2* (*p* = 0.0043), *Dkk1* (*p* = 0.0251) (Supplementary Fig. S2B–D). Activation of the JNK pathway was also suppressed by DR6 knockdown, indicated by reduced levels of phosphorylated JNK (*p* = 0.0064), as well as the mRNA level of *Jnk1* (Supplementary Fig. S2B, E). In the presence of Aβ_25-35_, DR6 downregulation further suppressed these two pathways (Fig. 7A–E), reflected by reduced levels of active β-catenin (*p* = 0.0051 versus Lenti-Ctrl+Aβ) and phosphorylated JNK (*p* = 0.0043 versus Lenti-Ctrl+Aβ) (Fig. 7A). We also observed a reduction in the mRNA expression of Wnt/β-catenin target genes (*Axin2*, *p* = 0.0197; *Dkk1*, *p* = 0.0109; *Notum*, *p* = 0.0436; all Lenti-shDR6 + Aβ versus Lenti-Ctrl + Aβ) and JNK signalling (*Jnk1*, *p* = 0.0312 Lenti-shDR6 + Aβ versus Lenti-Ctrl+Aβ) (Fig. 7B–E). In contrast, DR6 overexpression rescued the activation of Wnt/β-catenin and JNK signalling suppressed by Aβ_25–35_ treatment to a level similar to the control group (active β catenin, *p* = 0.0023; P-JNK, *P* = 0.0121; *Axin2*, *p* = 0.0155; *Dkk1*, *p* = 0.0146; *Notum*, *p* = 0.0016; *Jnk1*, *p* = 0.0136, all Lenti-DR6 + Aβ versus Lenti-Ctrl + Aβ) (Fig. 7F–J). However, overexpression of DR6 in the absence of Aβ_25__–__3__5_ did not affect activation of either Wnt/β-catenin or the JNK pathway (Supplementary Fig. S2F–I), as there were no significant change in the levels of active β-catenin (*p* = 0.6758 versus Lenti-Ctrl) and phosphorylated JNK (*p* = 0.9100 versus Lenti-Ctrl), as well as the mRNA expression of their target genes: *Axin2* (*p* = 0.9999) and *Dkk1* (*p* = 0.2681) in the Wnt/β-catenin pathway, although *Jnk1* (*p* = 0.0016) in the JNK pathway was upregulated (all versus Lenti-Ctrl, Supplementary Fig. S2F–I).

Taken together, these data demonstrate that the activation of Wnt/β-catenin and JNK signalling pathways is regulated by DR6 and Wnt/β-catenin/JNK signalling mediates the effect of DR6 on the BBB function [7]. Furthermore, increasing DR6 alleviates BEC dysfunction induced by Aβ through Wnt/β-catenin and JNK signalling.

## Discussion

**Fig. 8:**
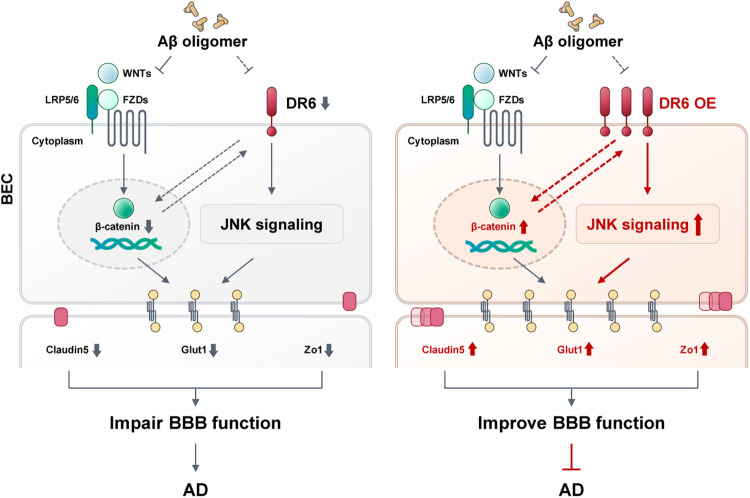
Upregulating brain endothelial DR6 restored Aβ-induced BBB malfunction in an in vitro model of Alzheimer’s disease. In Alzheimer’s disease, Aβ oligomers suppress death receptor 6 (DR6) in the brain BECs, leading to reduced endothelial functional proteins (Cldn-5, Zo-1, and Glut-1) and BBB malfunction through JNK signalling. At the same time, the Wnt/β-catenin pathway is also inhibited by Aβ oligomers, which interacts with DR6 to accelerate AD progression. Increasing DR6 expression in BECs can prevent Aβ-induced BBB malfunction by activating JNK and Wnt/β catenin signalling.

In this study, we demonstrated for the first time the role of brain endothelial DR6 in the pathogenesis of AD, particularly in maintaining BBB integrity in the presence of Aβ (Fig. 8). This endothelial angle differs significantly from prior understanding of the function of neuronal DR6 in AD. Therefore, our findings propose a new approach to developing interventions aimed at BBB in the context of AD. In contrast to common therapies aiming at reducing brain Aβ load, increasing DR6 specifically in the vasculature, can bolster the resilience of BECs at the BBB against the detrimental impact of Aβ and other pathological factors linked to AD. Thus, strengthening of BBB integrity offers protection against potential harm caused by circulating toxins and pathogens, representing a promising avenue for therapeutic intervention.

Here, we discovered the critical role of vascular-specific DR6 in AD-related BBB breakdown during early pathogenesis. We confirmed that DR6 is essential for maintaining normal levels of several key BBB functional proteins, such as Cldn-5 and Glut-1 [15, 16]. The decreased levels of these proteins are closely linked to early BBB breakdown, neuronal degeneration, and cognitive decline in AD patients [15, 16]. As expected, overexpressing DR6 in BECs rescued the levels of Cldn-5, Glut-1, and other BBB-related proteins, as well as BECs function in the presence of Aβ. These findings signify the therapeutic potential of BEC-specific increase in DR6 to protect BBB in AD brains. Incidentally, expression of DR6 is much higher in the brain vasculature, especially in endothelial cells, than in the other organs [7]. Physiologically, DR6 promotes angiogenesis in the developing brains and the production of tight junction proteins, as well as the establishment of BBB functions [7]. It needs to be noted that the overexpression of vascular DR6 only normalises tight junction proteins and endothelial function in AD-related pathological settings, but has no effect under non-pathological/healthy conditions. This can minimise the risk of angiomas.

Molecular pathways underlying the regulation of tight junction proteins in BECs by DR6 involve both Wnt and JNK pathways and also require the presence of Aβ. However, Aβ may act on DR6 to reduce JNK signalling, which downregulates tight junction proteins, rather than directly affecting JNK or junction protein expression. As a result, knocking down DR6 alone only resulted in limited inhibition of Cldn-5 and Glut-1 levels. However, in the presence of Aβ, we found a marked reduction in the vascular Wnt and JNK pathway elements after DR6 knockout. It seems that the cell type is the determining factor. Due to the critical role of JNK in regulating apoptosis, the overactivation of the neuronal JNK pathway has been closely linked to synaptic loss, Aβ depositions, and neuronal cell death [17]. Therefore, the therapeutic approach against this action is to knockdown JNK in neurons. On the contrary, our study showed that the intact or even increased Wnt/DR6/JNK signalling in the BECs is required to maintain and protect BBB against Aβ induced tight junction damage at the early stages of AD. This highlights the importance of avoiding generalisations of molecular pathways across the entire brain and instead underscores the need for specific segregation based on cell types.

Recent years witness growing interest on the role of microbiome pathogens, such as *Candida albicans* and *Corynebacterium tuberculosteriaticum*, in the pathogenesis of AD [18, 19]. Notably, the toxic molecules produced by these pathogens can disrupt the BBB, leading to abnormal glial function in the CNS, which further affect BBB integrity, ultimately contributing to the onset of AD [19]. During this process, it is conceivable that other unknown blood/organ-borne molecules induced by microbial toxins may also traverse a compromised BBB, further exacerbating AD-like pathology. Therefore, protecting BBB function is vital in preventing the brain from such risk factors.

In global DR6 knockout, the adverse impact on peripheral vascular integrity was not measurable as shown by the previous study [7]. This is advantageous as future treatment strategies targeting brain endothelial DR6 are unlikely to have peripheral vascular complications, such as increased microvessel resistance. As cognitive preservation in patients with AD can be independent of Aβ clearance, future studies can focus on the impact of BEC-specific upregulation of DR6 in vivo, in order to determine whether this approach can protect BBB and cognitive function in the context of Aβ accumulation during AD progression.

It is crucial to acknowledge that various cell types play a pivotal role in the architecture and functionality of the BBB, including pericytes and astrocytes. Endothelial cells, in particular, produce platelet-derived growth factor-BB. This growth factor acts through the platelet-derived growth factor receptor β on pericytes, facilitating the recruitment of pericytes around capillaries and thereby contributing to the stabilisation of blood vessels [20]. This interplay suggests a close relationship between endothelial cells and pericytes in the overall function of the BBB. Consequently, there is a need for future studies to delve into the involvement of DR6 in pericytes, examining its impact on BBB integrity and function.

## Conclusion

Our findings, summarised in Fig. 8, have shed light on the critical role of BEC DR6 in AD pathogenesis, particularly in relation to the BBB integrity. By enhancing vascular-specific DR6 expression, we offer a promising avenue for future interventions, which distinguishes itself from conventional strategies targeting Aβ aggregation. Moreover, the selectivity of DR6 in normalising tight junction proteins exclusively under AD-related pathological conditions has important implications, as it mitigates unintended complications. These collective insights not only advance our understanding of AD pathogenesis but also offer a hopeful direction for developing targeted therapies to combat this debilitating condition.

### Supplementary information

Supplementary Figure and table

Original Data File

## Data Availability

All datasets are presented in the main manuscript or additional supporting files.
