# Supplementary material for: Endothelial DR6 in blood-brain barrier malfunction in Alzheimer’s disease
Source: Cell Death Dis. 2024 Apr 12;15(4):258. doi: 10.1038/s41419-024-06639-0 (PMC11014957; doi:10.1038/s41419-024-06639-0)
Supplement: Supplementary file 1 — Supplementary Figure and table [file 41419_2024_6639_MOESM1_ESM.docx]

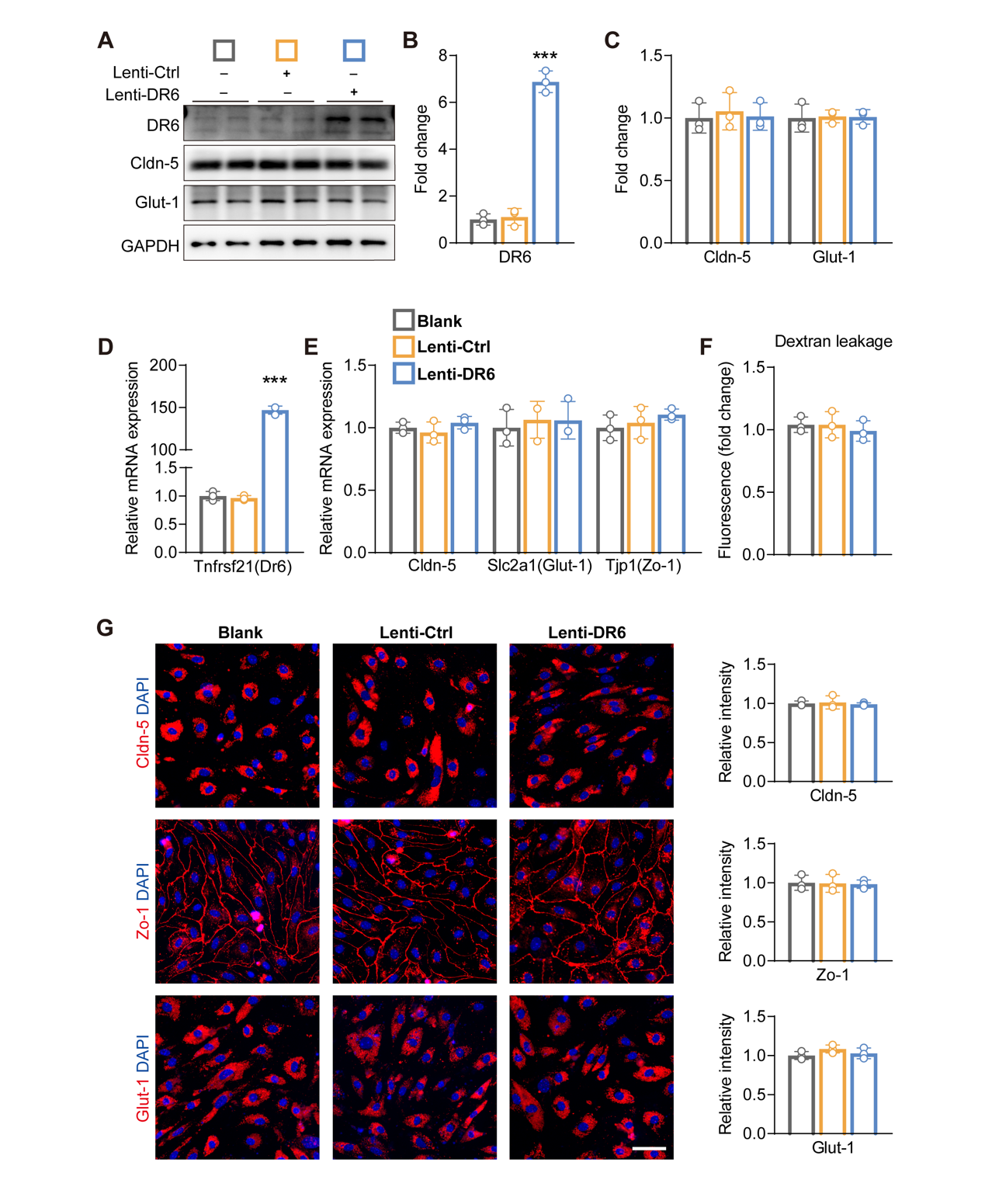


**Supplementary Fig 1. DR6 overexpression did not change BEC tight junction proteins and function.**

(A-C) Protein levels of DR6, Cldn-5, and Glut-1; (D-E) mRNA expression of *Dr6*, *Cldn-5*, *Glut-1*, and *Zo-1*; (F) *In vitro* trans-well permeability assay; (G) Immunostaining and quantification of Cldn-5, Zo-1, and Glut-1 in BECs (scale bar = 50 µm); Data are presented as mean ± SD, n = 3, *** p < 0.001.


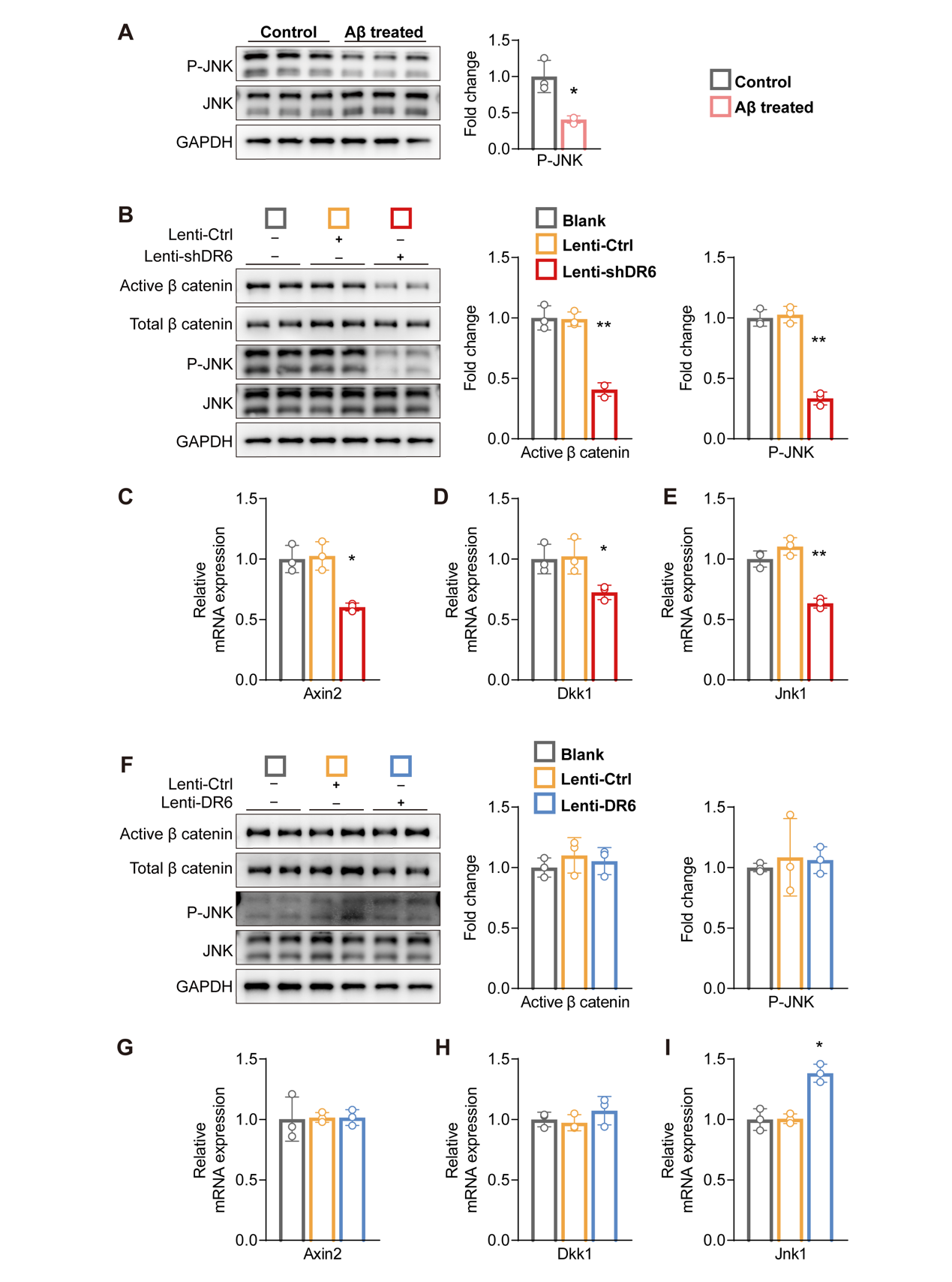


**Supplementary Fig 2.** **DR6 is required for Wnt and JNK signalling.**

(A) Protein levels of phosphorylated JNK; (B, F) Protein levels of active β-catenin and phosphorylated JNK; (C-E, G-I) mRNA expression of *Axin2*, *Dkk1*, and *Jnk1*. Data are presented as mean ± SD, n = 3, * p < 0.05, ** p < 0.01.

**Supplementary Table1.** **Primers used for real-time PCR**

| Gene Symbol | Forward primer (5->3) | Reverse primer (5->3) |
| --- | --- | --- |
| *Cldn-5* | ATGTCGTGCGTGGTGCAGAGT | GCGCCGGTCAAGGTAACAAAG |
| *Slc2a1(Glut-1)* | CACTGGTGTCATCAACGCCC | CACGGAGAGAGACCAAAGCG |
| *Tjp1(Zo-1)* | CGCTAAGAGCACAGCAATGG | TGGAGGTTTCCCCACTCTGA |
| *Axin2* | GCCGACCTCAAGTGCAAACTC | GGCTGGTGCAAAGACATAGCC |
| *Dkk1* | TTGACAACTACCAGCCCTACC | TCGGCAAGCCAGACAGATC |
| *Notum* | AACGTGGCACAGTTCCTTAT | CACACCCTCTAGTTCCCATTAC |
| *Jnk1* | ATGGCTGTCGATATTCAACCAG | CCTCTTGGGCATACCCCAC |
| *Tnfrsf21(Dr6)* | GCCATGTTGACCGTACCACT | CAGACTCGCAGGCTCATGTT |
| *Gapdh* | CCCCAGCAAGGACACTGAGCAA | GTGGGTGCAGCGAACTTTATTGATG |
