# Supplementary material for: Endothelial DR6 in blood-brain barrier malfunction in Alzheimer’s disease
Source: Cell Death Dis. 2024 Apr 12;15(4):258. doi: 10.1038/s41419-024-06639-0 (PMC11014957; doi:10.1038/s41419-024-06639-0)

Original blots for Fig 3C

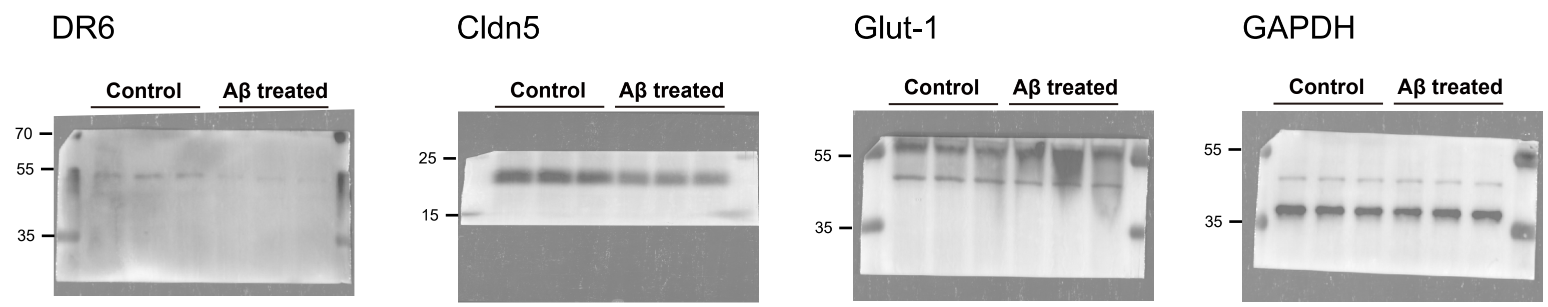

Original blots for Fig 3G

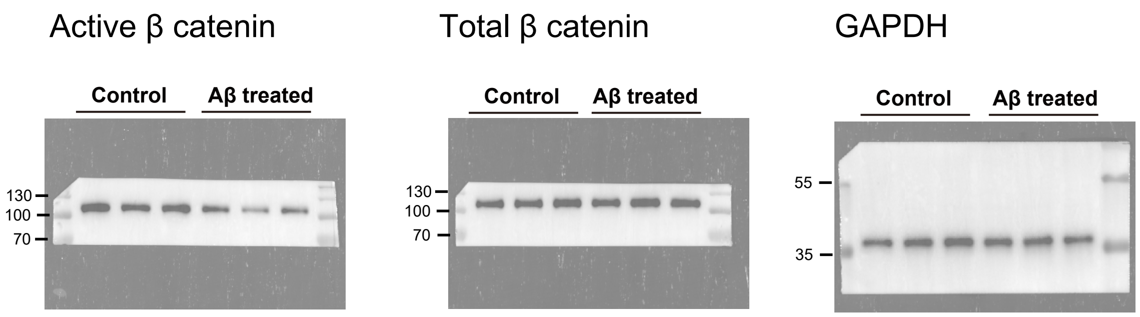

Original blots for Fig 4A

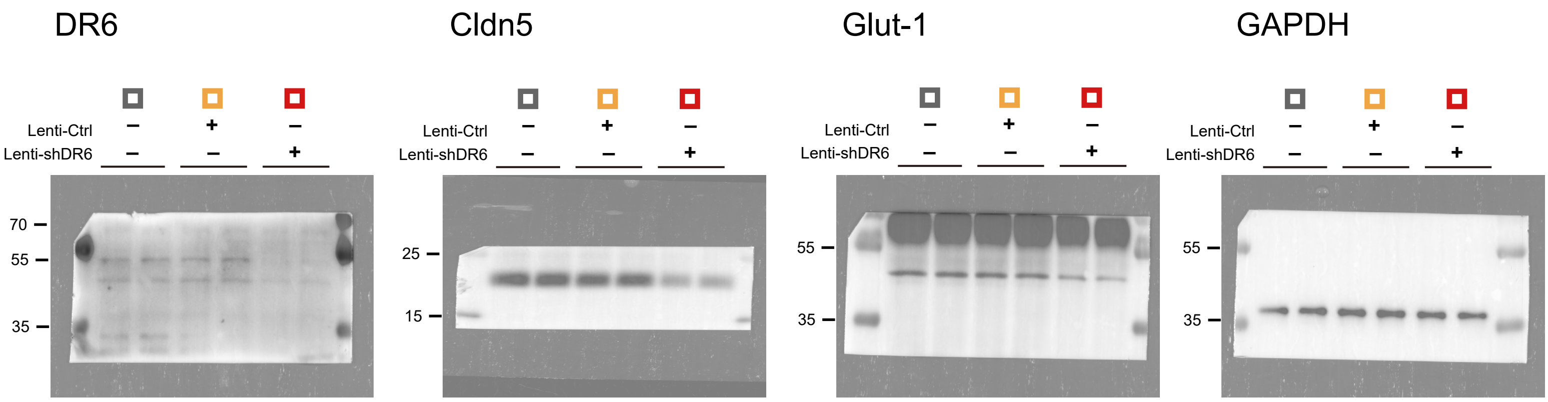

Original blots for Fig 5B

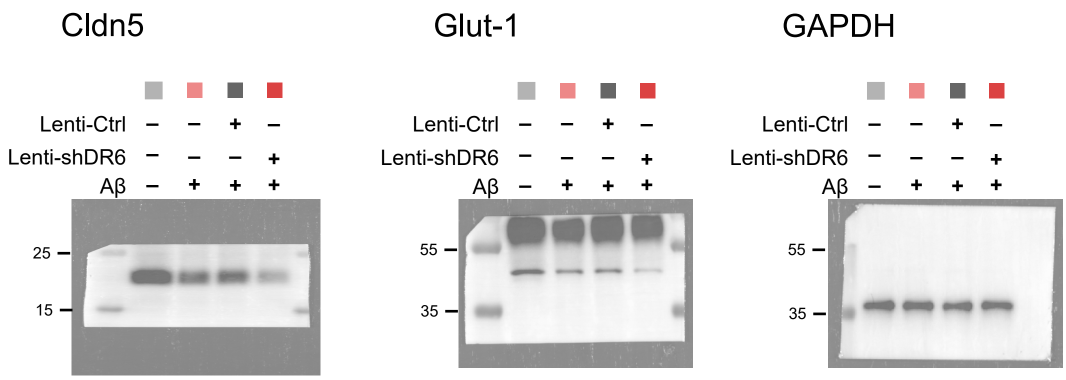

Original blots for Fig 6B

Cldn5

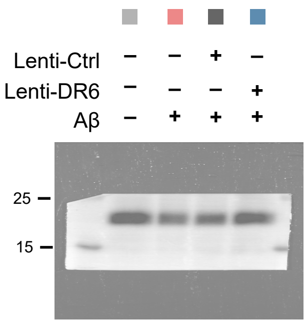

Glut-1

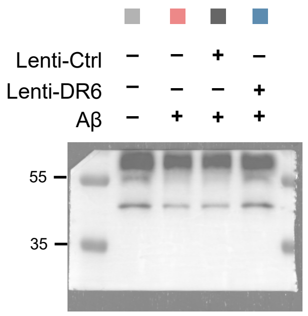

GAPDH

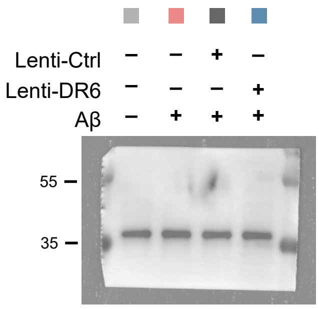

Original blots for Fig 7A

Active β catenin

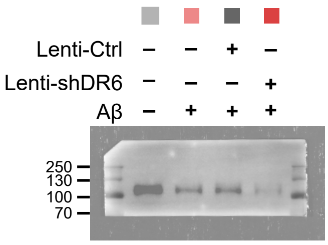

Total β catenin

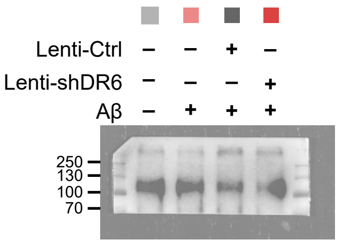

P-JNK

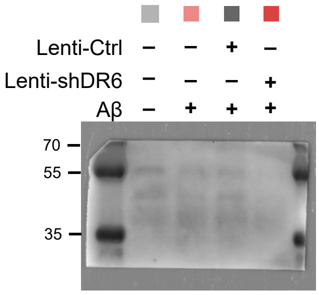

JNK

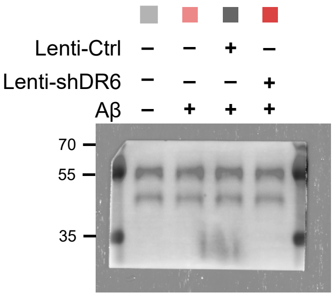

GAPDH

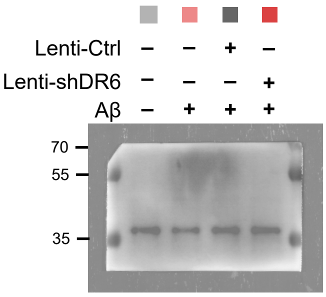

Original blots for Fig 7F

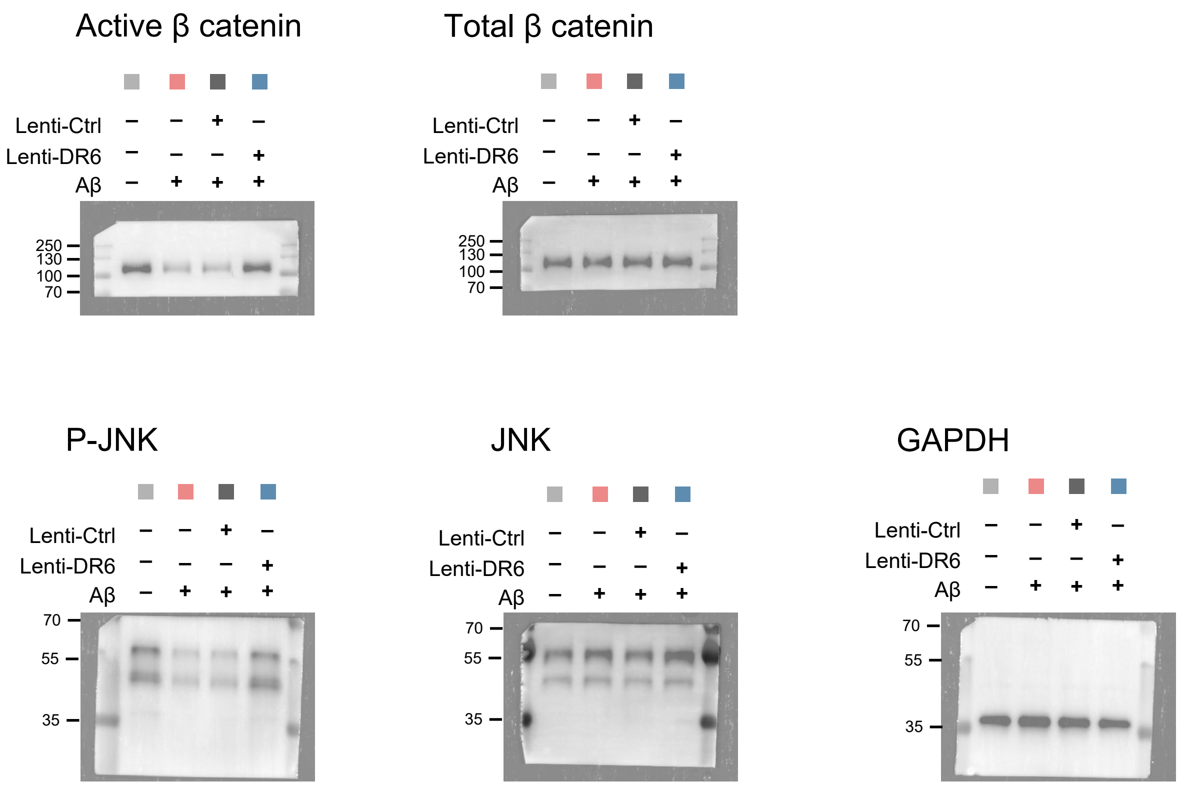

Original blots for Fig S1A

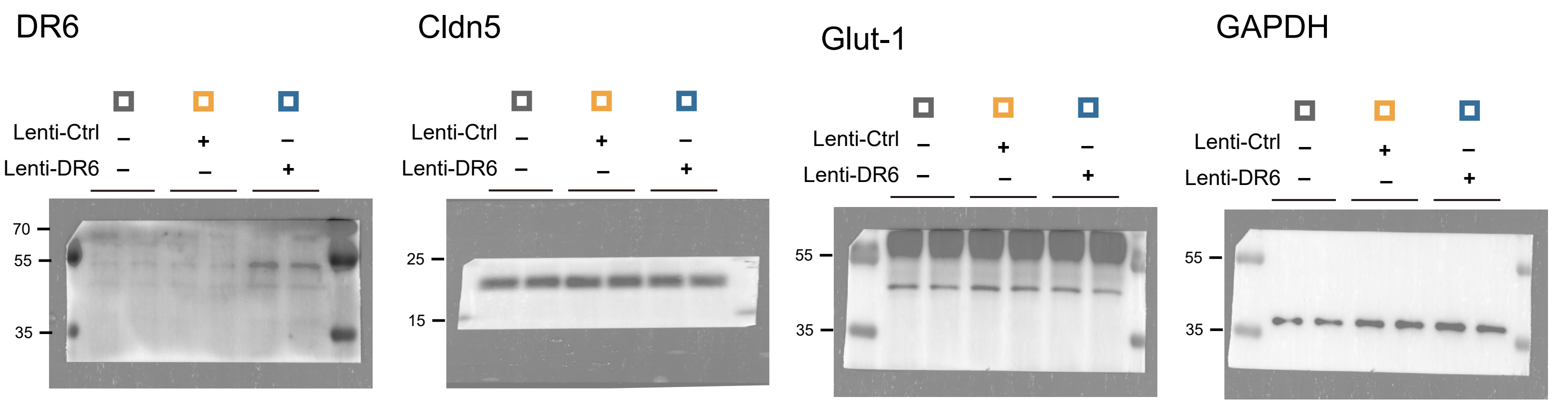

Original blots for Fig S2A

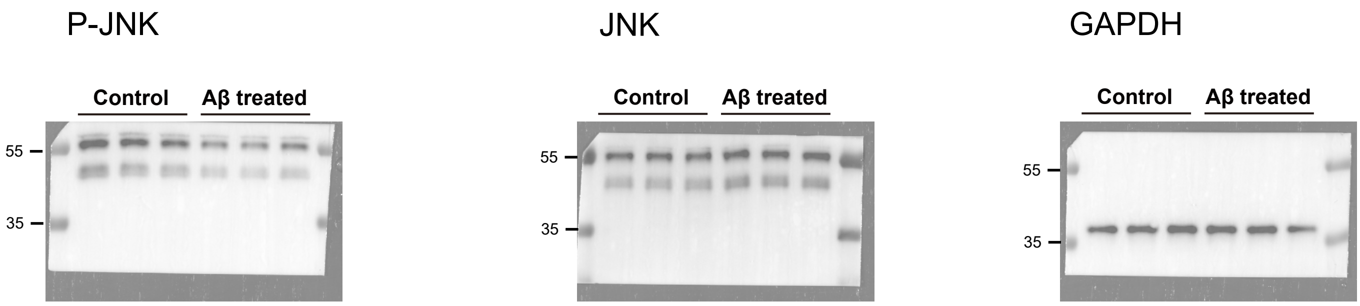

Original blots for Fig S2B

Active β catenin

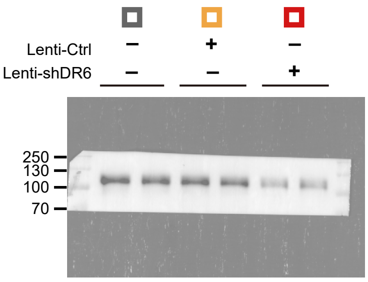

Total β catenin

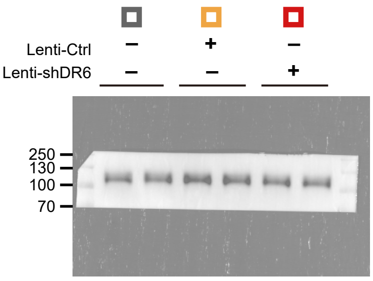

P-JNK

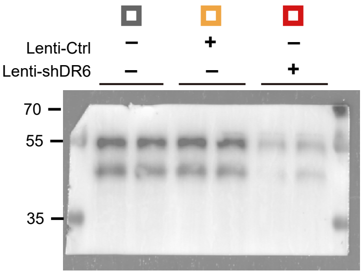

JNK

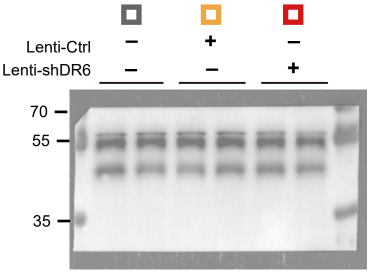

GAPDH

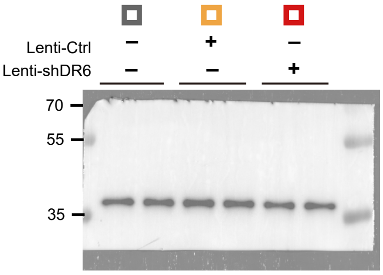

Original blots for Fig S2F

Active β catenin

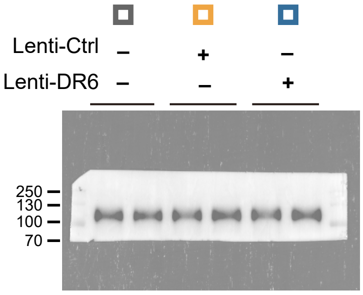

Total β catenin

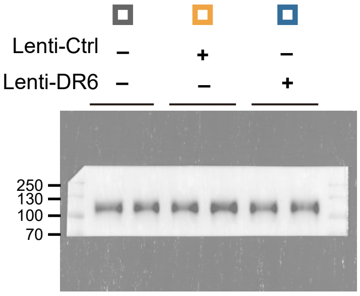

P-JNK

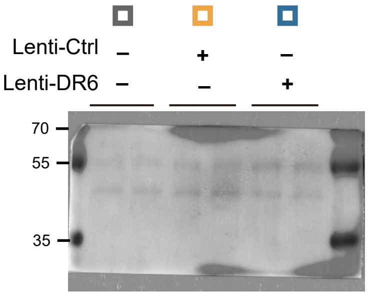

JNK

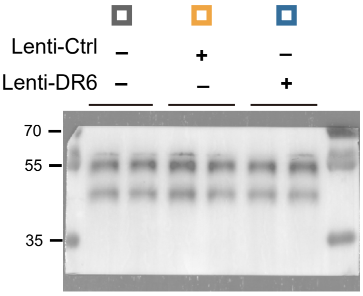

GAPDH

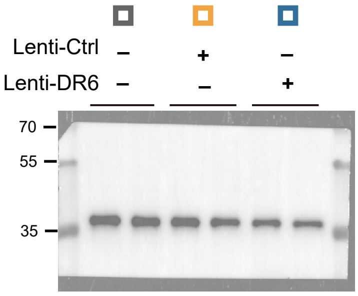

Supplement: Supplementary file 2 — Original Data File [file 41419_2024_6639_MOESM2_ESM.pdf]
